# Radiogenomics as an Integrated Approach to Glioblastoma Precision Medicine

**DOI:** 10.1007/s11912-024-01580-z

**Published:** 2024-07-16

**Authors:** Isabella Sanchez, Ruman Rahman

**Affiliations:** Biodiscovery Institute, School of Medicine, University of Nottingham, Nottingham, NG7 2RD UK

**Keywords:** Glioblastoma, Neuroimaging, Precision medicine, Deep learning, Radiomics, Radiogenomics

## Abstract

**Purpose of Review:**

Isocitrate dehydrogenase wild-type glioblastoma is the most aggressive primary brain tumour in adults. Its infiltrative nature and heterogeneity confer a dismal prognosis, despite multimodal treatment. Precision medicine is increasingly advocated to improve survival rates in glioblastoma management; however, conventional neuroimaging techniques are insufficient in providing the detail required for accurate diagnosis of this complex condition.

**Recent Findings:**

Advanced magnetic resonance imaging allows more comprehensive understanding of the tumour microenvironment. Combining diffusion and perfusion magnetic resonance imaging to create a multiparametric scan enhances diagnostic power and can overcome the unreliability of tumour characterisation by standard imaging. Recent progress in deep learning algorithms establishes their remarkable ability in image-recognition tasks. Integrating these with multiparametric scans could transform the diagnosis and monitoring of patients by ensuring that the entire tumour is captured. As a corollary, radiomics has emerged as a powerful approach to offer insights into diagnosis, prognosis, treatment, and tumour response through extraction of information from radiological scans, and transformation of these tumour characteristics into quantitative data. Radiogenomics, which links imaging features with genomic profiles, has exhibited its ability in characterising glioblastoma, and determining therapeutic response, with the potential to revolutionise management of glioblastoma.

**Summary:**

The integration of deep learning algorithms into radiogenomic models has established an automated, highly reproducible means to predict glioblastoma molecular signatures, further aiding prognosis and targeted therapy. However, challenges including lack of large cohorts, absence of standardised guidelines and the ‘black-box’ nature of deep learning algorithms, must first be overcome before this workflow can be applied in clinical practice.

## Introduction

Isocitrate dehydrogenase wild-type glioblastoma (GBM) is the most common and aggressive primary brain tumour in adults, comprising 49% of brain malignancy [1] with a dismal median survival rate of 14 months from diagnosis [2]. The current standard of treatment is maximal safe surgical resection, followed by concomitant radiotherapy and temozolomide chemotherapy [3]. GBM displays both intertumoral and intratumoral heterogeneity, which contributes to treatment resistance and subsequent tumour recurrence [4]. This is exacerbated by a high tumour proliferation rate and propensity to infiltrative brain parenchyma, which renders GBM incurable despite advances in treatment, and highlights the urgent need for a more individualised and targeted approach to patient care [5].

Imaging plays a vital role in diagnosis, treatment, and management of GBM by enabling physicians to visualise the tumour, aid surgical planning, and monitor tumour progression over the course of treatment. Conventional magnetic resonance imaging (MRI) is the standard, with myriad sequences used to provide high resolution structural information. However, conventional MRI has limitations: signals lack biological specificity, therefore limiting tumour characterisation; and it is limited in its ability to differentiate residual or recurrent tumour from treatment-related changes. Multiparametric MRI is a powerful tool that enables visualisation of diverse aspects of the tumour and its microenvironment [3]and enables clinicians to have a more accurate understanding of tumour progression throughout treatment, overcoming the intrinsic limitations of conventional MRI.

Radiomics is the emerging field of extracting quantitative data from medical images using advanced computer algorithms. It aims to capture texture-based and morphological features of tumours that cannot be appreciated with the human eye, and correlate these with clinical or biological endpoints [3]. Radiomics may overcome the issue of tumour heterogeneity, as it assesses the tumour in its entirety, as well as the tumour habitat, lesion margins, and surrounding peri-tumoral regions, including oedema subcompartments [3]. It therefore offers promise in precision oncology, and in future clinical decision-making.

The most impactful application of radiomics in neuro-oncology is likely to be realised in radiogenomics. This is an area of research investigating the relationship between radiomic features and the respective underlying genomic landscape and provides a mechanism for establishing statistical associations between both datasets. Radiogemomics aims to derive molecular characterisation of the tumour, such as gene expression profiles, epigenetic marks, or genetic mutations, based on the tumour’s radiophenotype. Recent investigative studies have identified several driver mutations that are reported to have prognostic and predictive implications in GBM tumours. There is potential for these to be used as specific targets for personalised therapies; however, current practice (tissue biopsy) means molecular profiling is prone to sampling bias due to the size of the sample being unable to capture the heterogeneity of the tumour. In providing a phenotype for a tumour corresponding to its genotype, radiogenomics may allow for overcoming the limitations of tumour biopsy, and aid in personalising treatment decisions [6].

Here, we review recent advancements in the use of MRI radiogenomics for the assessment of molecular markers of interest in GBM regarding prognosis, response to treatments, monitoring recurrence, and survival prognostication. Such an appraisal will facilitate consideration of the future possibility of using imaging biomarkers alone to identify molecular targets for GBM precision therapy.

### Advanced MRI Techniques

Neuroimaging plays a vital role in the diagnosis, prognosis, and treatment of GBM. The clinical standard is conventional MRI, which consists of two fundamental sequences: pre- and post- gadolinium T1-weighted imaging (T1w), and T2-weighted fluid attenuated inversion recovery (T2-FLAIR)[7]. T1w MRI enhances the signal of fatty tissue and suppresses that of water and is the current standard for defining the tumour burden at initial diagnosis. However, conventional MRI is insufficient in providing the detail required in complex and heterogenous disease conditions such as GBM. There is therefore an urgent clinical need for more accurate and comprehensive imaging modalities, and for these to be made widely available, for clinicians to gain a more thorough comprehension of a patient’s condition, thus providing optimal care.

Advanced MRI techniques allow for a more complete understanding of the tumour microenvironment, including tumour metabolism and haemodynamics, vascular permeability, and cellular proliferation. These techniques are more sensitive to the different biophysical processes within tissues, and therefore provide a more comprehensive insight. Combining these techniques to construct a multiparametric scan enables visualisation of diverse aspects of the tumour and its microenvironment by combining several MRI techniques into a single scan.

### Diffusion Techniques

#### Diffusion-Weighted Imaging

Diffusion-weighted imaging (DWI) is based upon the random movement of water molecules within biological tissues, which follows the principles of Brownian motion, caused by intermolecular collisions. Apparent Diffusion Coefficient (ADC) is used to measure the magnitude of this random molecular movement, with factors such as cellular packing, the presence of intracellular organelles, cell membranes, and macromolecule content, contributing to this measurement [8]. This quantitative parameter can reflect cellular membrane integrity and tissue cellularity, aiding the visualisation of tumour boundaries. Variations in these values may be attributed to the alteration and redistribution of water molecules between intracellular and extracellular tissue compartments [8].

Cellular density and tumour grade are directly related to the degree of water restriction on DWI [8]. This results in an inverse correlation between tumour grade and ADC values in GBM; thus, high-grade tumours, accommodating densely packed cancerous cells and diminished extracellular space, exhibit low ADC values. Changes in ADC values of a tumour may prove useful in monitoring tumour response, as it is expected that in successful concurrent chemotherapy (which results in necrosis and cellular lysis), tumour cellularity would be reduced, and therefore ADC values would increase.

#### Diffusion Tensor Imaging

Diffusion tensor imaging (DTI) is a more advanced method of diffusion imaging. This is a mathematical model of diffusion in 3D space, consisting of a 3 × 3 matrix derived from diffusivity measurements in at least six nonplanar directions, with utilisation of greater than six measurements increasing tensor measurement accuracy [9]. This method allows a constant description of the shape of water diffusion regardless of rotation, enabling DTI to be applied to the complex fibre tracts in the brain, and therefore enhancing our knowledge of intracranial structural connectivity.

GBM is known to preferentially spread along white matter tracts, through a complex process of adhesion, motility, and invasion, where infiltrative disease cells induce pathologic demyelination and vasogenic oedema [9]. The sensitivity of DTI to changes in diffusion of water in white matter tracts can aid identification of defects in these tissues, which appear to correlate with tumour spread, and which cannot be observed using conventional MRI scans. Additionally, simplification of this data into isotropic and anisotropic components allows the production of tissue ‘signatures,’ which can distinguish between infiltrated and normal (non-infiltrated) white matter[10].

### Perfusion Techniques

Tumour neovascularisation is correlated with tumour grade, and inversely correlated with positive outcomes. Perfusion techniques measure how well a tissue is supplied with blood and is currently the most accurate method in differentiating between tumour progression and pseudoprogression.

#### Dynamic Susceptibility Contrast

Dynamic susceptibility contrast (DSC) is the most commonly used method of perfusion weighted imaging and involves the intravenous administration of a bolus of gadolinium-based contrast agent, followed by a series of rapidly acquired gradient-echo images over the brain [8]. This method relies on a transient drop in signal intensity due to the transit of gadolinium through vasculature during its initial pass. It allows calculation of relative cerebral blood volume (rCBV) within a region of interest, in addition to other perfusion parameters, such as cerebral blood flow (the volume of blood passing through a given region of tissue per unit time) and mean transit time [11]. There is a strong correlation between elevated rCBV and GBM; therefore, these parameters could aid surveillance imaging of low-grade gliomas to monitor for early signs of progression and transformation to a high-grade glioma.

#### Dynamic Contrast Enhanced

Dynamic contrast enhanced T1w- perfusion MRI imaging is an alternative method that relies on T1 changes in tissues over time after bolus administration of gadolinium-based contrast agent. It utilises pharmacokinetic modelling that typically requires an additional T1 mapping protocol to generate tissue perfusion parameters and can therefore be used as a measure of vascular permeability [11]. Limitations of DSC include lower temporal resolution compared to DSC, and a lack of consensus for the best pharmacokinetic model.

#### Combining Diffusion and Perfusion Parameters

Spatial and temporal intratumoral heterogeneity within GBM means that the use of a single imaging technique or parameter is not always reliable in its characterisation and in evaluating treatment response. The multiparametric method in which several quantitative MRI techniques are analysed in combination can be used to address this challenge. Diffusion and perfusion imaging techniques provide distinct yet complementary physiological information; therefore, it may be assumed that in using both techniques and associated data analysis, they act synergistically, providing enhanced diagnostic power than if either imaging modality was analysed alone [8]. Studies have reported that merging of these parameters can aid assessment of tumour invasiveness, prediction of survival, and evaluation of GBM response to immunotherapy, suggesting that this multiparametric approach may provide a more accurate evaluation of a patient’s condition [6, 12].

## Artificial Intelligence and Machine Learning in Radiology

**Fig. 1:**
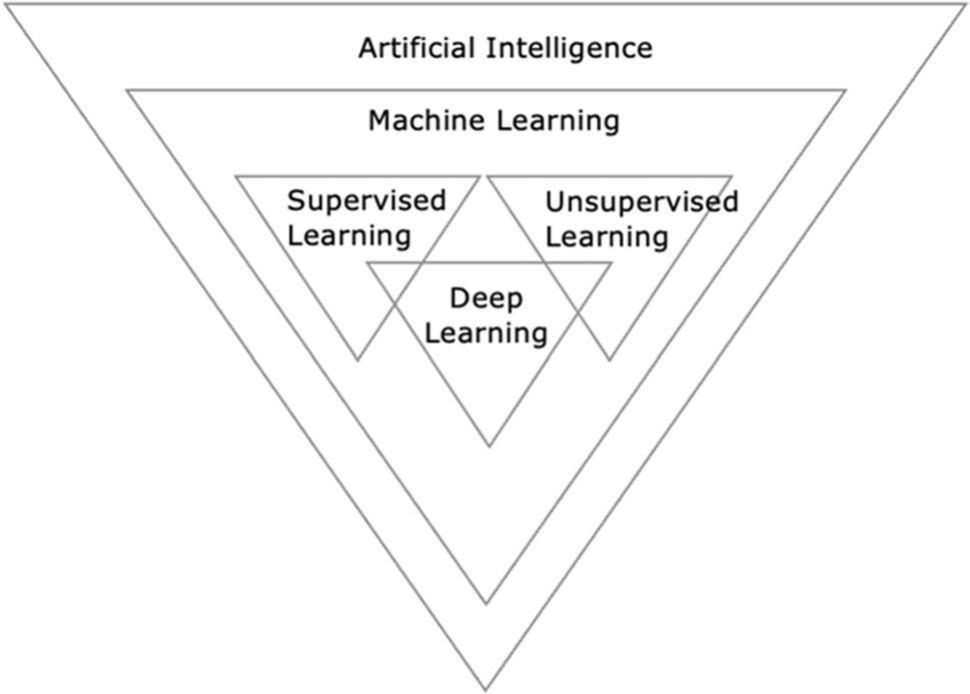
Branches of Artificial Intelligence. Artificial Intelligence (AI) is the general term used to define a task performed by a computer that models human intelligence or behaviour. Machine Learning (ML) is a branch of AI in which models are trained to learn by experience, identify patterns, and then make predictions based on new data. ML can be divided into supervised algorithms, which are trained on a human-labelled dataset; and unsupervised models, which are trained on unlabelled data. Deep Learning (DL) is a further subfield of ML that utilises multi-layer neural networks to learn from large sets of data and make predictions. It can also be divided into supervised and unsupervised methods

Machine Learning (ML) is a branch of Artificial Intelligence (AI) in which analytical algorithms and statistical models are trained on sample data to learn by experience, identify patterns, and then make predictions based on new data. This can be further divided into supervised and unsupervised algorithms. Supervised ML algorithms are trained on a human-labelled dataset (Fig. 1). This makes it useful for predictive modelling, with patient traits used as input data, and the outcome of interest as the output. These patient traits can range from baseline data, such as age and gender, to more specific disease-based information, such as gene expression, clinical symptoms, and diagnostic imaging [13]. The goal of this algorithm is to predict a known output or target on new, unlabelled, independent data, and does so through classification (data is assigned into specific categories) and regression (used to understand the relationship between the dependent and independent variables). Unsupervised ML in contrast, identifies undisclosed patterns in unlabelled independent datasets that are unrecognised by humans [14]. This ability to elucidate hidden structures in data renders unsupervised ML a promising approach to feature extraction, thus useful in image detection, classification, and segmentation within medical imaging [15].

**Fig. 2:**
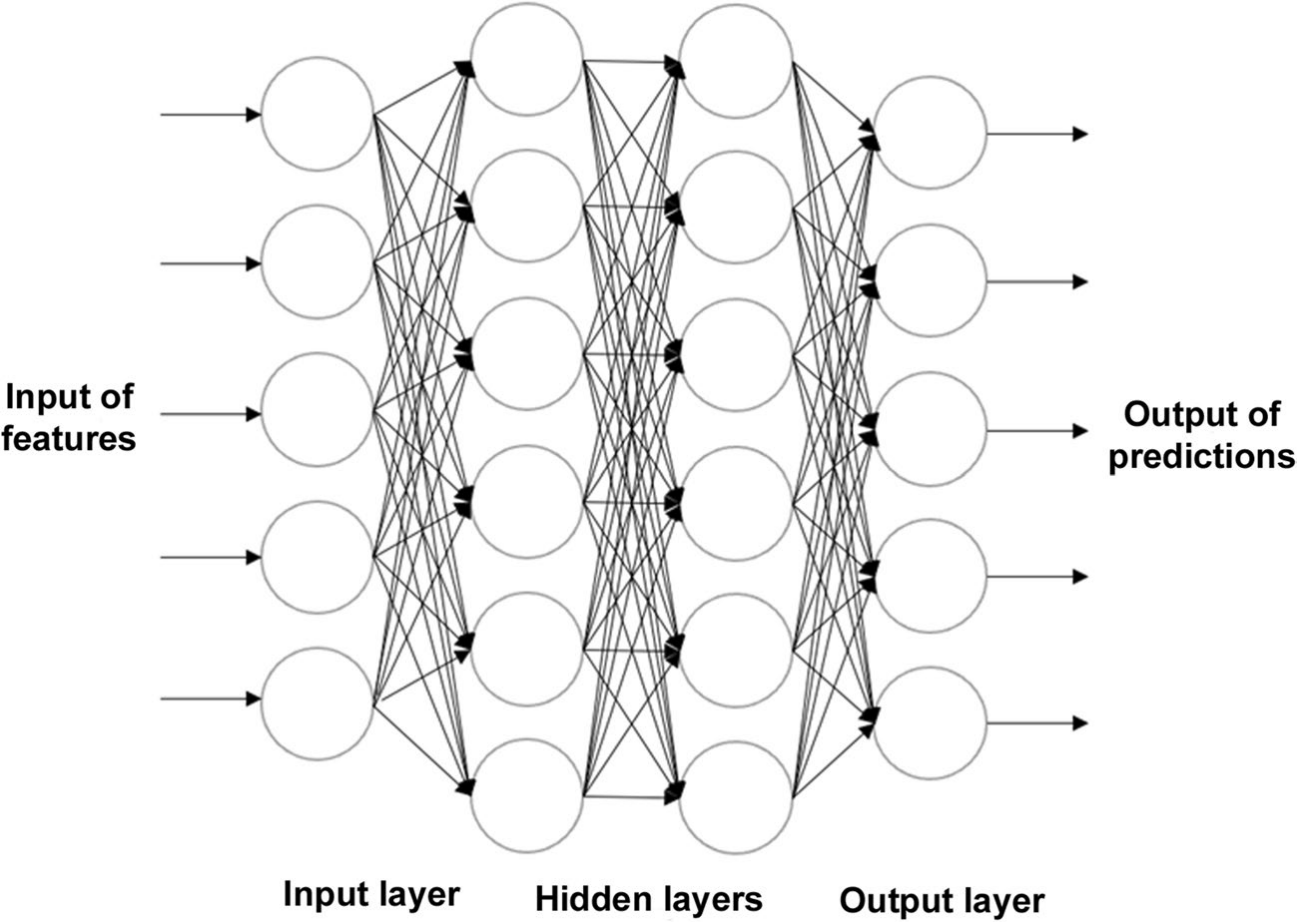
Visualisation of Deep Neural Network Architecture (Multiple Layers). Deep Learning Neural Networks aim to imitate the neuronal signaling of the human brain. These neural networks are comprised of multiple layers of interconnected nodes. Each layer builds upon the previous layer to enhance predictions. The input and output layers are ‘visible layers,’ and the layers between these are ‘hidden layers,’ as they depend on a previous input of data that is unknown to humans

Deep Learning (DL) is a further subfield of ML that is characterised by the operation of Artificial Neural Networks (Fig. 1). These networks are inspired by the human brain, and the way in which neuronal signalling occurs. Multiple datasets are analysed at the same time, and these are evaluated and processed multiple times. Each evaluation occurs on a different layer, which consists of nodes (units) that are connected to both the previous and the subsequent layers [15]. Each node applies an activation function to transform the data in a nonlinear manner, to refine and optimise the prediction or categorisation. The input and output layers are known as ‘visible layers,’ with the layers between these termed ‘hidden layers,’ as they depend on a previous input of data that is not visible. This training process allows deeper layers in the network to combine high‐level features generated from prior features and enables building more such features iteratively [16, 17] (Fig. 2).

## Glioblastoma Radiomics

Radiomics is defined as the extraction of information from routine radiological scans, and the transformation of these textural and morphological tumour characteristics into quantitative data [14]. Feature-based radiomics extracts a set of mathematically predefined features from a region-of-interest (ROI).

### Image Pre-Processing

Pre-processing is the process of standardising images that were acquired using different protocols, through image transformation. It therefore ensures reproducibility and generalisability of the extracted features and generated models, especially if the data is acquired from different scanners across multiple participating healthcare sites. Pre-processing can also be used to account for patient movement within the scanner. Typical algorithms which are used, include pixel resampling, intensity standardisation, and noise reduction [14, 18, 19].

### Tumour Segmentation

Inter-rater variability has been recognised as a factor that can reduce the reliability of the radiomic features, and therefore confound the accuracy and interpretation of analysis [3]. The development of ML algorithms to automatically detect and segment GBM, demonstrate potential to increase the efficiency of this step.

### Feature Extraction

Algorithms that capture tumour heterogeneity across local pixel neighbourhoods are used to extract features from ROI for radiomic analysis[20]. Radiomic features evaluate certain characteristics of an image using a numerical value, essentially providing a quantifiable summary of a ROI on a radiologic image. These can be categorised into four subgroups:

i. *Shape features:* These describe the geometric properties of the ROI, such as volume or surface area. [21].
ii. *First order features:* These consider the pixel values of a ROI using the image intensity distribution represented by histograms [20]
iii. *Second order features:* These relate to the spatial distribution of pixel values and quantify intratumoral heterogeneity. [20].
iv. *Higher order features:* These are computed from first- or second-order features usually after the application of image transformations, such as filtering or wavelet transformations [3].

### Feature selection

Correlations between features and relevant biological and clinical outcomes are identified. Feature selection can use either univariate or multivariate statistical models and are classified into supervised and unsupervised methods [14] The most commonly used supervised models include:

i. *Filter models (univariate):* These assess the correlation between labels and features but do not consider redundancy [20].
ii. *Wrapper methods (multivariate):* A predictive model is used to score the performance of a subset of features, aiming to identify the subset that results in the best performant model [14].
iii. *Embedded methods:* These perform the feature selection process within the construction of the model itself, are less prone to overfitting the data, more accurate than filter methods, and faster than wrapper methods [20].

### Challenges in Developing Models for Clinical Applications

A key difficulty in developing radiomics models within neuro-oncology is the inability to obtain sufficient data to ensure accuracy. Multi-centred collaborations are therefore needed to build larger datasets for appropriately powered training and validation of models, and which are made publicly accessible [22]. Radiomics is also currently limited in its generalisability due to variations in image acquisition protocols across various scanners and institutions. Differences in factors such as image contrast, slice thickness, voxel resolutions and magnetic field strengths all contribute to this disparity [18, 22].

The method of segmentation is another inconsistency between radiomic studies. While some studies use automatic and semiautomatic methods, many use manually contoured lesions [22]. Although widely considered the highest standard, this is more time-consuming and labour-intensive, and can introduce observer bias, high inter-reader variability, increased variability in image acquisition, and the derivation of inconsistent radiomic features.

Although there is potential for DL-based radiomics to overcome these limitations regarding differences in protocols, a unique set of challenges arise. DL features are considered a ‘black-box,’ meaning that they arrive at conclusions without any explanation as to how this was achieved; the internal mechanisms and contributing factors of DL-based radiomics models remain unknown [14]. In addition, there is relative sparsity of training samples for these models in general, which becomes even more apparent when focussing on relatively rare cancers such as GBM, where obtaining large datasets may not be feasible [22].

## Glioblastoma Radiogenomics

Treatment for GBM largely remains ineffective due to its heterogenous nature. Current classification of GBM is mainly based on histologic features of the tumour, which does not reflect the genetic and molecular differences between both tumours in individual patients, and within each tumour. Recent advancements in genomic technology have enabled a better understanding of these alterations. Genomics, therefore, has the potential to contribute to GBM management and molecular pathology in multiple ways, from aiding clinical distinction between primary and recurrent tumours, to the identification of mutations that are favourable for targeted, personalised treatments.

**Fig. 3:**
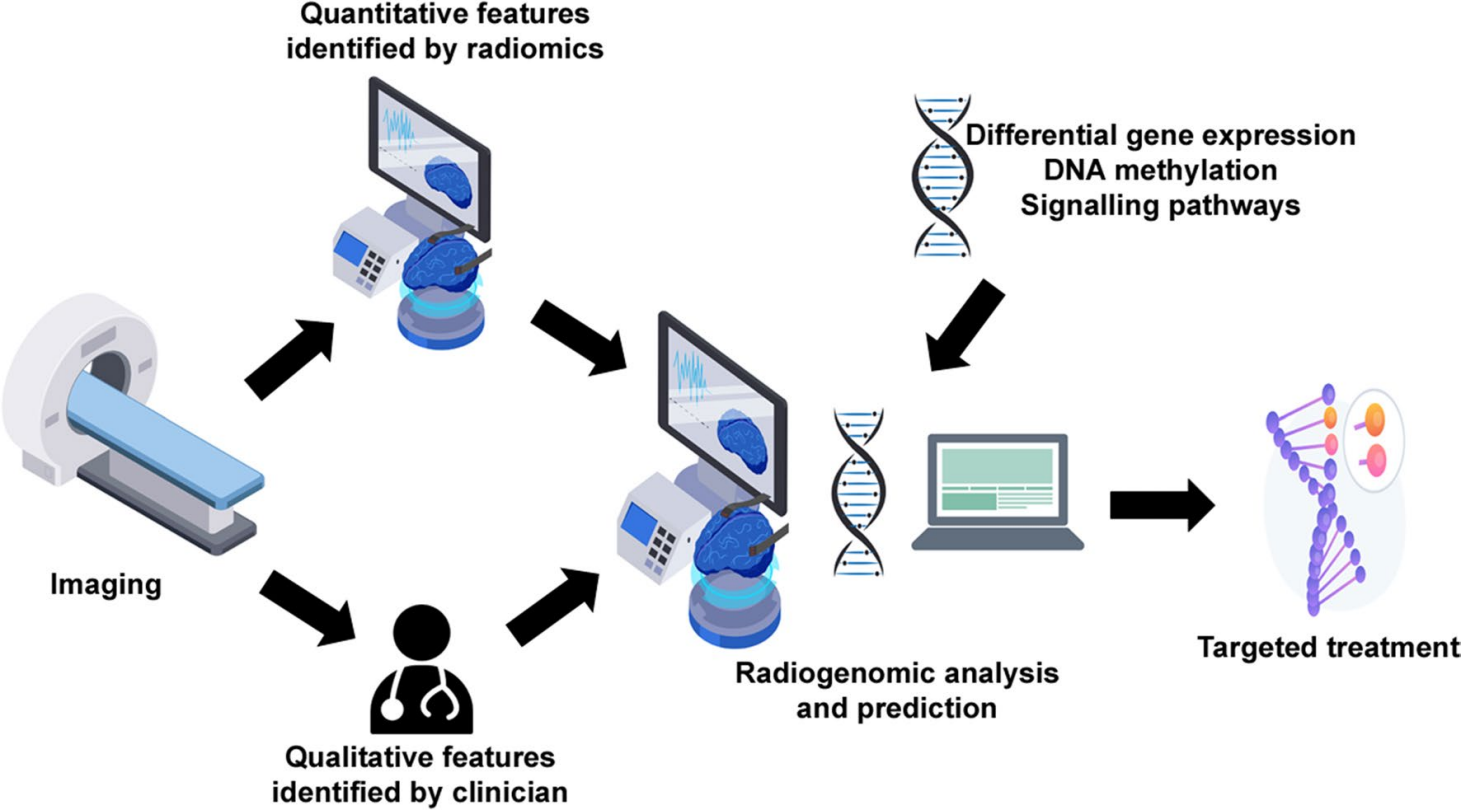
Radiogenomics Workflow. Radiogenomics links quantitative imaging features with GBM genomic profiles. The workflow involves imaging of a patient’s brain, followed by identification of both qualitative features (by a clinician) and quantitative features (through radiomics). Trained models are then able to create predictions on the tumour’s genotype based on this information, enabling a more targeted treatment to be delivered to the patient. Adapted from [24] Created with Freepik

Radiogenomics is a specific application of radiomics, in which imaging features are linked with such genomic profiles. It is a powerful means of studying GBM, and has exhibited its ability in characterising GBM, predicting molecular signatures, and determining therapeutic response and survival of newly diagnosed patients [14] (Fig. 3).

Radiogenomic studies are either exploratory (aim to establish relationships between tumour radiographic characteristics and gene expression profiles) or hypothesis-driven (based on the assumption of variations of genes, molecular-subtypes, or pathways, through exploration of relevant radiophenotypes that best characterise the anticipated genomic alteration) [23].

### Isocitrate Dehydrogenase Mutational Status

The isocitrate dehydrogenase enzyme converts isocitrate to ketoglutarate, which leads to the production of nicotinamide adenine dinucleotide phosphate (NADPH). Whilst GBM is characterised by isocitrate dehydrogenase wild-type status, the presence of isocitrate dehydrogenase mutation characterises WHO grade 4 astrocytoma which typically results in a less aggressive disease, with a more favourable prognosis, and an increase in both overall and progression-free survival (relative to isocitrate dehydrogenase wild-type tumours)[23]. In 2016, WHO recommended the addition of isocitrate dehydrogenase status to the molecular classification of gliomas due to its significant prognostic value in stratification.

Numerous studies have suggested associations between isocitrate dehydrogenase mutation and tumour location. Altieri et al. (2018) reported that isocitrate dehydrogenase mutant tumours more commonly occurred on the right hemisphere, with wild-type tumours having the highest incidence in the temporal lobe [25]. The most promising revolution in the field, is the application of DL algorithms. Choi et al. (2021) utilised MRI data from a range of centres to predict isocitrate dehydrogenase genotypes in glioma patients using an automated approach [26]. Two convolutional network models (CNN) models were designed: one for segmentation, and another for isocitrate dehydrogenase status prediction. An excellent diagnostic accuracy of 93.8% and 87.9% in internal and external data sets respectively, was obtained. This fully automated process establishes a highly reproducible and generalisable means to noninvasively predict isocitrate dehydrogenase status of GBM and grade 4 astrocytoma.

### O.^6^-methylguanine-DNA Methyltransferase (MGMT)

The MGMT gene encodes the DNA repair enzyme, O^6^-methylguanine-DNA alkyltransferase. Methylation of the MGMT promotor is associated with an enhanced response to alkylating agents such as temozolomide, increased response to radiotherapy, and increased overall survival [27]. However, achieving accurate prediction is challenging, as MGMT promotor methylation is irregular, area-specific, and can change over the disease course [3]. Numerous studies have aimed to predict MGMT methylation status using a variety of imaging modalities. Wei et al. (2019) extracted radiomic features from T1w, T2-FLAIR, and ADC maps, and developed a fusion radiomics signature to combine clinical factors with these texture features [28].

DL algorithms have also been investigated to aid this prediction. Korfiatis et al. (2017) compared three different residual deep neural network (ResNet) architectures to evaluate their ability to predict MGMT methylation status in GBM patients [29]. ResNet50 (50 layers) outperformed the other models with shallower architectures (ResNet34 and ResNet18, with 34 and 18 layers respectively), achieving an accuracy of 94.9% in the test set. More recently, a DL pipeline has been utilised for automatic tumour segmentation and MGMT promotor status classification using T1w and T2-FLAIR images [30].

### Biochemical Signalling Pathways

Radiogenomics studies have identified relationships between different imaging features and key aberrant cell signalling pathways. Liu et al. utilised quantitative imaging features extracted from preoperative perfusion MRI to identify an angiogenic subgroup of GBM patients, in which pathways related to angiogenesis and hypoxia are activated [31]. Enhanced perfusion was significantly associated with poor patient survival. In 2020, Park et al. showed that their diffusion- and perfusion-weighted MRI radiomics model could successfully predict core signalling pathways of receptor tyrosine kinase, p53 and retinoblastoma in IDH wild-type GBM [32]. Inclusion of physiologic MRI and clinical parameters improved the accuracy of these predictions.

Alterations to these signalling pathways can also affect a tumour’s response to treatment. Beig et al. (2018) found that Law’s energy features (features quantifying the presence of edges, spots, and ripples from the enhancing region on T1w-MRI) could accurately predict GBM response to chemo-radiation treatment and were significantly correlated with Protein Kinase B (AKT) and apoptosis signalling pathways.

Epidermal Growth Factor Receptor (EGFR) is a transmembrane tyrosine kinase which regulates cell division and death [23]. The most common mutant of EGFR in GBM is EGFRvIII, expressed in 31% of patients [33], and overexpression or amplification of EGFR is a feature of aggressive primary GBM and associated with tumour infiltration [34]. Using MRI, Aghi et al. (2005) reported a T2w-to-T1w enhancement ratio for tumours (likely reflecting increased angiogenesis and oedema), in addition to fuzzier tumour borders (reflecting increased invasiveness) in those with EGFR overexpression [35]. Using multi-parametric MRI and a support vector machine-based approach, Akbari et al. (2018) composed an imaging signature of EGFRvIII in GBM patients, which exposed a complex yet distinct GBM phenotype [36]. The model achieved an 87% accuracy, with the signature consistent with these tumours having increased neovascularisation and cell density, thus being typical of a more infiltrative phenotype.

Collectively, this use of MRI features to noninvasively identify alterations in GBM core signalling pathways may further support the development of next-generation targeted therapy.

### Molecular Subtypes

In 2010, Verhaak et al. revealed a gene-expression based classification of GBM, namely classical, neural, proneural and mesenchymal subtypes [37]. These were found to respond differently to aggressive therapies, thus resulting in varying survival benefits and aiding patient stratification for molecular targeted therapy. Differences in imaging phenotypes amongst the different molecular subtypes have been demonstrated in radiogenomic studies. The proneural subtype, more commonly found in younger patients, is associated with a better prognosis [38], and the mesenchymal subtype is considered the most aggressive [39]. Volume of contrast enhanced (CE) tumour, volume of central necrosis (CN), combined volume of CE and CN, and the ratio of T2-FLAIR to CE and necrosis, were significantly different in mesenchymal GBM compared to non-mesenchymal GBM. These four metrics were all identified as significant predictors of the mesenchymal subtype, with the volume ratio of T2-FLAIR hyperintensity to CE and CN also capable of stratifying short- and long-term overall survival [40]. Furthermore, the combination of rCBV parameters with molecular subtypes has been shown to increase the predictive performance of models [41].

### Heterogeneity

Radiogenomics has shown promise for spatial evaluation of the distinct regional and genetic subtypes that coexist within a single tumour. Hu et al. (2017) co-registered biopsy locations with multiparametric MRI and texture maps to establish a relationship between regional genetic status with spatially matched imaging measurements [42]. Significant imaging correlations were identified for six driver genes, including EGFR, RB1 and TP53, supporting the application of image-based biomarkers to aid the characterisation of intratumoral heterogeneity, and thus, offer diagnostic value to precision oncology.

### Immunophenotypes

Hsu et al. (2020) utilised radiomic features and a ML-based radiomics model to classify the immunophenotypes of GBM and predict patient prognosis [43]. Furthermore, using multiparametric MRI and a consistent clustering method, Lin et al. (2020) classified radiological phenotypes into two subgroups, observing significant differences in tumour immune cell infiltration and immunotherapy biomarkers between these, and concluded that different GBM subtypes respond differently to immunotherapy, an insight valuable in treatment planning [44]. Immune cell markers have also been reported to have significant correlations with perfusion and diffusion MRI features including ADC, further confirming their potential in prediction of prognosis and progression in GBM [45].

### Challenges

One of the fundamental challenges for radiogenommic integration into clinical practice is the necessity of large, standardised data sets. External validation is a crucial step in confirming model generalisability, yet many studies lack this due to limited data. The requirement for substantial amounts of data becomes even more apparent with the integration of DL methods, as sufficiently large numbers and diversity cannot easily be acquired from a single institution alone [27]. The Cancer Imaging Archive (TCIA), which provides open-access resources on cancer imaging and corresponding genomics, is however, insufficient. Increased data sharing between institutions nationally and internationally will allow further development in this field.

Furthermore, variation in imaging protocols across various scanners and sites (including image contrast, voxel resolutions, slice thicknesses and repetition times) influences radiomic features, and consequently the predictive models. Tumour habitat segmentation methodology varies, with some studies using manual segmentation (introducing high inter-observer variability), whilst others used automatic segmentation methods (inconsistencies between existing segmentation algorithms introduces variability) [14]. Standardisation of the radiogenomic workflow (image acquisition, preprocessing, segmentation, and radiomic feature extraction pipelines) across large multi-site cohorts is therefore vital to improve generalisability, repeatability, and reproducibility.

Despite the introduction of DL networks offering a range of opportunities for GBM research, features and models generated by AI often lack interpretable parameters and are considered a ‘black-box’ [15]. They possess neither a set of diagnostic rules, nor any insight into the generated results. In order for radiogenomic methods that utilise AI to be widely accepted in clinical practice, visualisation of models and features must first be established.

## Future Directions and Conclusion

Genetic sequencing of a tumour is crucial in the precision medicine workflow. The identification of key genetic signatures such as isocitrate dehydrogenase and EGFR, and the success of models to predict these, highlights the potential for radiogenomics in diagnosis, prognosis, and treatment of GBM. Early prediction and understanding of tumour biology is essential to maximise effectiveness of GBM treatment, and radiogenomic methods can enable this. The volume of information generated from these individual markers is immense, and the combination of these markers even more powerful.

Although DL models have been developed to predict MGMT promotor methylation, achieving remarkable success [29], further radiogenomic models should be created to predict methylation status of other segments of tumour DNA. Tonicity-responsive enhancer binding protein (NFAT5) for example, has recently been reported to be overexpressed and correlated with poor outcomes in GBM. Li et al., (2023) concluded that NFAT5 lysine methylation results in TMZ resistance and targeting this methylation may provide a therapeutic strategy for GBM treatment [46]. Another example introduces long non-coding RNAs (lncRNAs), which play important roles in tumorigenesis and cell cycle regulation. Lu et al. (2020), revealed that abnormally low methylation of lncRNA SNHG12 plays an important role in TMZ resistance, and is correlated with poor overall survival and drug sensitivity in a clinical setting [47]. Creation of DL-based algorithms to determine such genome-wide methylation status could therefore offer advances in GBM prognosis and treatment.

Radiogenomic studies have identified variations in imaging phenotypes between different GBM subtypes (gene-expression based classification) [40], and recognised four metrics as significant predictors of the more aggressive mesenchymal subtype. Thus, in future practice, this imaging process may in principle, be used to circumvent genomic profiling and permit commencement of therapy (predicated on molecular targets enriched within the mesenchymal subtype) more rapidly, resulting in improved outcomes.

GBM pathogenesis involves complex alterations at genetic, transcriptional, proteomic, and metabolic levels [48]. In depth analysis of these molecular changes are imperative to enable a more comprehensive characterisation of a tumour’s biology and heterogeneity. It is likely that in the future, the radiomics field will evolve and differentiate into subdivisions such as ‘radiotranscriptomics,’ ‘radioproteomics’ and ‘radiometabolomics.’ Multi-omics integration analysis combining this information from different molecular levels will provide a holistic view of tumour behaviour [48]. Integration of AI and DL-based models to this multi-omics approach will provide further advancements to precision medicine in order to aid development of more precise therapeutic strategies.

These radiogenomic studies leave us with the provocative question: can imaging alone be used to identify molecular targets for therapy? Challenges including lack of large cohorts, absence of standardised multi-institutional guidelines and the ‘black-box’ nature of DL algorithms, must first be overcome before this workflow can be applied in clinical practice. However, the rapid rate of development in the radiogenomics field in recent years provides confidence that this non-invasive method will play a significant role in both the management of GBM and patient prognosis in the future.

## Data Availability

No datasets were generated or analysed during the current study.
